# Assessment of the Impact of Electronic Prescribing on Antimicrobial Stewardship in an Inpatient Old Age Psychiatric Setting

**DOI:** 10.1192/bjo.2025.10623

**Published:** 2025-06-20

**Authors:** Wan Yee Ko, Vanessa Tolulope Jacobs, Monira Miah, Roberta Manuel, Ivan Shanley

**Affiliations:** Essex Partnership University NHS Foundation Trust, Rochford, United Kingdom

## Abstract

**Aims:** Infections are common amongst old age psychiatry patients in an inpatient setting, which often require treatment with antimicrobials. Antimicrobial stewardship is important in reducing side effects caused by unnecessary antimicrobial use as well as preventing antibiotic resistance.

Electronic prescribing is becoming increasingly common in clinical practice. Reasons supporting these systems include increasing efficiency of prescribing and reducing human error. The old age psychiatry ward in Rochford Hospital migrated from paper drug charts to electronic prescribing (ePMA) from the beginning of December 2024. ePMA allows prescribers to set an end date or a review date for each prescription, which may help reduce the number of unnecessary or inappropriately prolonged duration of antimicrobial prescriptions.

This study aimed to compare antimicrobial prescriptions made on paper drug charts to those on ePMA.

**Methods:** Two groups of patients were included in the study. One group consisted of 28 patients admitted prior to December 2024 who had their medications prescribed on paper charts, including 14 instances of antimicrobial prescriptions. The paper charts were retrieved from electronic patient records. The other group (n=38) had electronic prescribing available with 25 antimicrobial prescriptions. The parameters reviewed included the duration of antimicrobials, documentation of an end date or review date, and whether an indication was stated for the prescription.

**Results:** Prescriptions on paper charts included end dates in 71% of cases, whereas this fell to 48% on ePMA (p=0.03). Of those without end dates on paper, 100% had no review date, compared with 62% on ePMA. Paper prescriptions included indications for treatment in half of cases, whereas no indications were documented on ePMA (although indications were consistently documented in the medical notes).

**Conclusion:** Antimicrobial prescriptions on paper drug charts were more likely to have had a set duration compared with those on ePMA, with a statistically significant fall in the presence of end dates following the introduction of ePMA. Indications for treatment were also more accessible on paper charts. Limitations of the paper charts were noted through the process however, for example paper charts may be replaced prior to a new prescription being written, meaning the information might not be as readily available as this study suggests. Overall, it is clear that prescribers will need to adjust to ePMA to ensure good antimicrobial stewardship. This might be achieved via simple dialogue boxes as prompts.

